# A Combination of Flaxseed Oil and Astaxanthin Improves Hepatic Lipid Accumulation and Reduces Oxidative Stress in High Fat-Diet Fed Rats

**DOI:** 10.3390/nu9030271

**Published:** 2017-03-13

**Authors:** Jiqu Xu, Shuang Rong, Hui Gao, Chang Chen, Wei Yang, Qianchun Deng, Qingde Huang, Lingyun Xiao, Fenghong Huang

**Affiliations:** 1 Department of Nutriology, Oil Crops Research Institute, Chinese Academy of Agricultural Sciences, 2 Xudong Second Road, Wuhan 430062, China; xujiqu@foxmail.com (J.X.); qchun2@126.com (Q.D.); viperson1973@163.com (Q.H.); 2 Hubei Key Laboratory of Lipid Chemistry and Nutrition, Oil Crops Research Institute, Chinese Academy of Agricultural Sciences, 2 Xudong Second Road, Wuhan 430062, China; 3 Key Laboratory of Biology and Genetic Improvement of Oil Crops, Ministry of Agriculture, 2 Xudong Second Road, Wuhan 430062, China; 4 Department of Nutrition and Food Hygiene, School of Public Health, Medical College, Wuhan University of Science and Technology, No. 2, Huangjiahu Road, Wuhan 430065, China; shuang_rong@yeah.net; 5 Department of Nutrition and Food Hygiene, School of Public Health, Tongji Medical College, Huazhong University of Science and Technology, 13 Hangkong Road, Wuhan 430030, China; gaohuitj@yeah.net (H.G.); yangw1983@foxmail.com (W.Y.); 6 Department of Gastroenterology, The First People’s Hospital of Yichang, The People’s Hospital of China Three Gorges University, 2 Jiefang Road, Yichang 443000, China; chenchang1976@163.com; 7 Department of Gastroenterology, The People’s Hospital of China Three Gorges University, 2 Jiefang Road, Yichang 443000, China; 8 Functional Oil Laboratory Associated by Oil Crops Research Institute, Chinese Academy of Agricultural Sciences and Infinite (China) Co., LTD., 66 Jianzhong Road, Guangzhou 510665, China; Xiaolingyun12@126.com

**Keywords:** flaxseed oil, astaxanthin, high fat diet, lipid accumulation, oxidant stress

## Abstract

Hepatic lipid accumulation and oxidative stress are crucial pathophysiological mechanisms for non-alcoholic fatty liver disease (NAFLD). Thus, we examined the effect of a combination of flaxseed oil (FO) and astaxanthin (ASX) on hepatic lipid accumulation and oxidative stress in rats fed a high-fat diet. ASX was dissolved in flaxseed oil (1 g/kg; FO + ASX). Animals were fed diets containing 20% fat, where the source was lard, or 75% lard and 25% FO + ASX, or 50% lard and 50% FO + ASX, or FO + ASX, for 10 weeks. Substitution of lard with FO + ASX reduced steatosis and reduced hepatic triacylglycerol and cholesterol. The combination of FO and ASX significantly decreased hepatic sterol regulatory element-binding transcription factor 1 and 3-hydroxy-3-methylglutaryl-CoA reductase but increased peroxisome proliferator activated receptor expression. FO + ASX significantly suppressed fatty acid synthase and acetyl CoA carboxylase but induced carnitine palmitoyl transferase-1 and acyl CoA oxidase expression. FO + ASX also significantly elevated hepatic SOD, CAT and GPx activity and GSH, and markedly reduced hepatic lipid peroxidation. Thus, FO and ASX may reduce NAFLD by reversing hepatic steatosis and reducing lipid accumulation and oxidative stress.

## 1. Introduction

Nonalcoholic fatty liver disease (NAFLD), which includes benign hepatic steatosis and cirrhosis, has been shown to be associated with obesity, hyperlipidemia and type II diabetes [1,2]. As the prevalence of these diseases is increasing worldwide, NAFLD has become a serious health concern [3]. NAFLD and associated increased prevalence of coronary artery disease (CAD) [4] contribute to more morbidity and mortality and create greater health-care costs. The pathogenesis of NAFLD is thought to occur via a “double-hit”: hepatocellular lipid accumulation and subsequent oxidative stress [5].

*N*-3 polyunsaturated fatty acids (*n*-3 PUFAs) have been of interest because of their purported benefits for many chronic diseases including cardiovascular disease, cancer, and type II diabetes [6]. The plant-derived *n*-3 PUFA, α-linolenic acid (ALA), is nutritionally essential and cannot be synthesized de novo in vertebrate tissue [7]. ALA can compete with linoleic acid to reduce arachidonic acid or act as a precursor for longer chain *n*-3 PUFAs (such as EPA and DHA), or it can directly interact with nuclear receptors and ion channels to produce positive effects [8]. Because it is high in ALA, flaxseed oil (FO) may be a promising functional food ingredient. Recently, ALA has been shown to lower hepatic lipids and reduce NAFLD [9]. However, ALA is particularly susceptible to oxidation, so addition of FO may increase hepatic lipid peroxidation [10], which may adversely affect hepatoprotection.

As a lipophilic xanthophyll carotenoid, astaxanthin (ASX) is produced de novo by microorganisms and plants, but it is also found in diverse microalgae, fungi and crustaceans. ASX has a hydroxyl (OH) and keto (CdO) moiety on each ionone ring and its molecular structure lends it powerful antioxidant activity [11]. Recently, ASX has been studied for its anti-inflammatory, anti-tumor, anti-diabetic, cardiovascular and neuroprotective activities as well for its ability to modulate immune function [11,12,13,14,15]. ASX has been shown to protect against hepatic endoplasmic reticular stress, inflammation and lipid deposition in high fat, high-fructose fed mice [16,17], as well as protect against dioxin-induced hepatotoxicity [18]. However, the effectiveness of administration of antioxidants alone is suboptimal to inhibit the progression of NAFLD [19]. We previously reported that FO and ASX reduced plasma oxidative stress, lipids, and inflammation, and may contribute to cardiovascular protection [20]. Thus, we studied whether this combination can reduce liver damage in rats fed a high-fat diet.

## 2. Methods

### 2.1. Chemical Sources

Flaxseed oil was obtained from Caoyuankangshen Food Co., Ltd. (Inner Mongolia, China). Commercial deodorized lard was purchased from a local supermarket. Astaxanthin was obtained from Jingzhou Natural Astaxanthin Inc. (Jingzhou, China) and was dissolved in flaxseed oil to a final concentration of 1 g/kg (FO + ASX) when used.

### 2.2. Animals and Diets

Forty male Sprague–Dawley rats (initially 150–170 g) were purchased from Sino-British Sippr/BK (Shanghai, China). Animals were housed individually and maintained at a controlled ambient temperature (22 ± 1 °C) under diurnal conditions (light–dark: 08:00–20:00) with access to laboratory chow and tap water *ad libitum*. After 1 week of acclimatization, rats were randomized into a high-fat diet (CON) group and three experimental groups (*n* = 10 animals/group). All animals were fed purified experimental diets as shown in Table 1. Dietary fat was provided by lard (CON group), or 75% lard and 25% FO + ASX (L-FO + ASX group), or 50% lard and 50% FO + ASX (M-FO + ASX group), or 100% FO + ASX (H-FO + ASX group). Weekly purified diets were mixed, formed into a dough with purified water, rolled into pellets, sealed in air-tight plastic bags under nitrogen gas and stored at −80 °C until use. Food in the cages was shaded from light and changed daily. All animals were weighed twice a week and food intake was measured weekly. Animals were cared for in accordance with The Guiding Principles in the Care and Use of Animals. The experiment was approved by the Oil Crops Research Institute Council on Animal Care Committee, Chinese Academy of Agricultural Sciences ((2013) IACUC Number: 0021).

### 2.3. Tissue Preparation

After 10 weeks of treatment, rats were fasted for 16 h and then killed under anesthesia. Then, cardiac blood was collected with sodium heparin and centrifuged at 1500× *g* for 10 min at 4 °C to remove plasma which was stored at −80 °C. Livers were rapidly dissected, weighed, and a small piece of the right liver lobe was fixed in 4% paraformaldehyde for light microscopy. Remaining liver tissue was stored at −80 °C until analysis.

### 2.4. Serum Assays

Plasma aspartate aminotransferase (AST) and alanine aminotransferase (ALT) were quantified using a Hitachi 7020 auto-analyzer with commercial kits (Wako Pure Chemicals, Osaka, Japan).

### 2.5. Liver Histology

Liver was sectioned at 5 μm thickness and stained with hematoxylin and eosin (H&E). Fifteen digital images per animal were selected for estimating volume density (Vv) of liver steatosis by point-counting hepatic fat droplets as previously described [21].

### 2.6. Liver Lipid Content

Lipids were extracted from 1 g liver with a mixture of chloroform/methanol (2:1, *v*/*v*) by the method of Folch [22]. Hepatic triglyceride (TG) and total cholesterol (TC) were measured with commercial kits (Zhongsheng Beikong Biotech Company, Beijing, China).

### 2.7. Western Blot

Liver samples were lysed in ice-cold RIPA buffer supplemented with 1 mM PMSF (Sigma, St. Louis, MO, USA) and then kept at 4 °C for 2 h. Supernatant was collected by centrifuge at 10,000× *g* for 15 min at 4 °C. For SDS–PAGE, samples were mixed with SDS sample buffer and incubated at 98 °C for 5 min. Western blot was performed with the following antibodies: β-actin mAb (#3700, Cell Signaling, Danvers, MA, USA), peroxisome proliferator activated receptor α (PPARα) pAb (ab8934, Abcam, Cambridge, UK), sterol regulatory element-binding transcription factor 1 (SREBP1) mAb (sc-365513, Santa Cruz Technology, Santa Cruz, CA, USA), and 3-hydroxy-3-methylglutaryl-CoA reductase (HMGCR) mAb (ab174830, Abcam).

### 2.8. Quantitative Real-Time PCR

Total RNA of the liver was isolated using Trizol (Invitrogen, Grand Island, NY, USA) as indicated by kit instructions. Using the RT system (Takara Bio, Dalian, China), cDNA was synthesized from total RNA. Real-time PCR was performed with SYBR Premix Ex Taq II (Takara Bio, Dalian, China), using the ABI 7900HT real-time thermocycler (Applied Biosystems, Forster, CA, USA). The dissociation curve of each gene was performed and analyzed using ABI 7900HT software, and the result confirmed product specificity. Each sample was analyzed three times and normalized to β-actin. Results of real-time PCR were analyzed with the 2^−ΔΔCt^ method as previously described [23]. Primer sequences for genes in this study were as follows. fatty acid synthase (FAS): forward 5′-GGACATGGTCACAGACGATGAC-3′, reverse 5′-GTCGAACTTGGACAGATCCTTCA-3′; acetyl CoA carboxylase (ACC): forward 5′-GCCTCTTCCTGACAAACGAG-3′, reverse 5′-TCCATACGCCTGAAACATGA-3′; carnitine palmitoyl transferase-1 (CPT-1): forward 5′-AACTTTGTGCAGGCCATGATG-3′, reverse 5′-AGCTTGTGAGAAGCACCAGCA-3′; acyl CoA oxidase (ACO): forward 5′-ATCTCTGTGGTTGCTGTGGAGTCA-3′, reverse 5′-TCTGGATGCTTCCTTCTCCAAGGT-3′.

### 2.9. Assay of Hepatic Antioxidant Activity and Lipid Peroxidation

Livers were weighed and a 10% homogenate was prepared in a 50 mmol/L phosphate buffer (pH 7.0) containing 0.1 mmol/L EDTA. Homogenate was centrifuged at 1000× *g* for 10 min at 4 °C for subsequent tests. Superoxide dismutase (SOD) activity was measured according to the method of Kono [24]. Glutathione peroxidase (GPx) activity was assayed by the method of Sazuka [25]. Catalase (CAT) activity was estimated using the method of Goth [26]. Glutathione (GSH) was quantified using methods published by Moron [27]. Thiobarbituric acid reactive substances (TBARS) were quantified using the method of Buege [28], and all methods used have been previously described [29].

### 2.10. Protein Measurement

Protein was quantified using the Lowry method [30] and bovine serum albumin (BSA) as a standard.

### 2.11. Statistical Analyses

Results were expressed as means ± SEM (standard error of the mean), and analyses were based on one-way ANOVA, followed by the Fisher PLSD *post hoc* test if differences were significant. All statistical analyses were performed using SPSS 13.0 statistical software (SPSS Inc., Chicago, IL, USA), and the limit of statistical significance was set at *p <* 0.05.

## 3. Results

### 3.1. Food Intake and Body Weight Gain

There were no differences in food intake and weight gain among all groups during the study period (data not shown).

### 3.2. Effects of FO and ASX Combination on Hepatic Enzymes in Plasma

**Figure 1:**
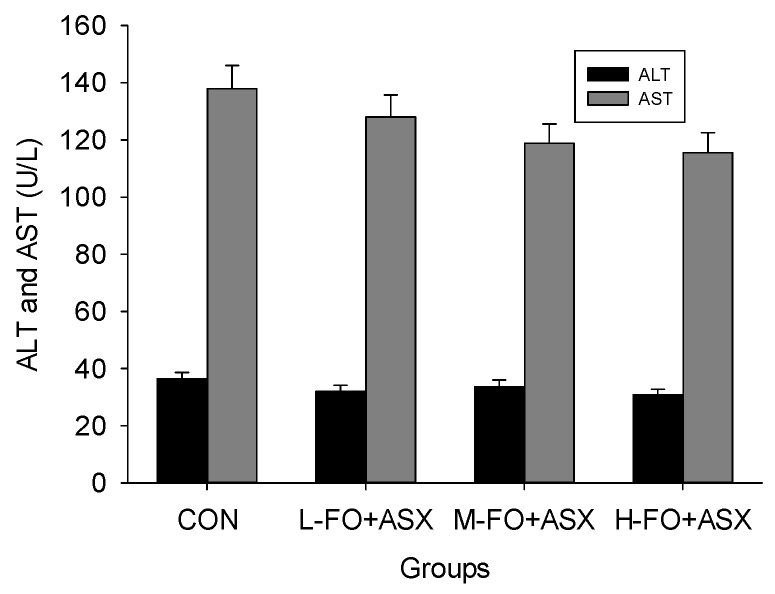
Effects of flaxseed oil (FO) and astaxanthin (ASX) on plasma alanine aminotransferase (ALT) and aspartate aminotransferase (AST) in rats fed a high-fat diet. CON: high-fat diet group; L-, M- and H-FO + ASX: low, moderate, and high FO and ASX groups. Bars represent means ± SEM (*n* = 10/group).

Plasma ALT and AST decreased in all FO + ASX groups (Figure 1), but these changes never reached statistical significance (*p* > 0.05).

### 3.3. Effects of FO and ASX on Liver Morphology

**Figure 2:**
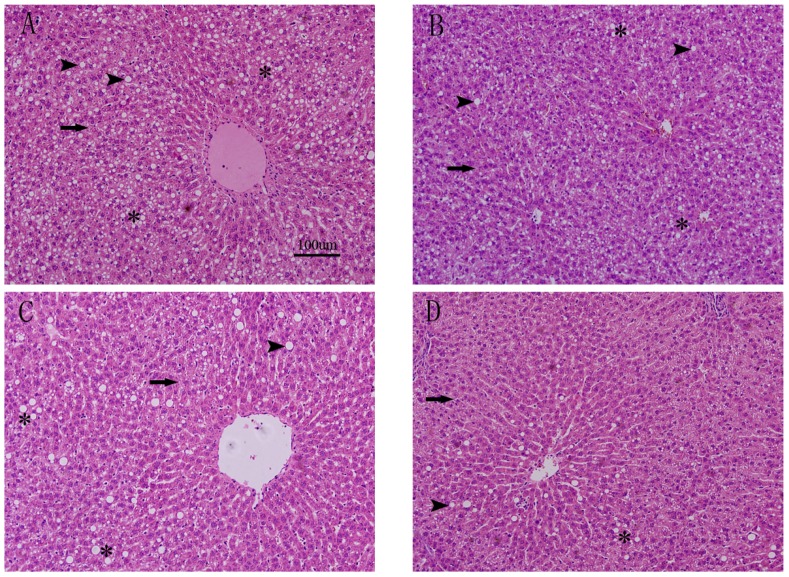

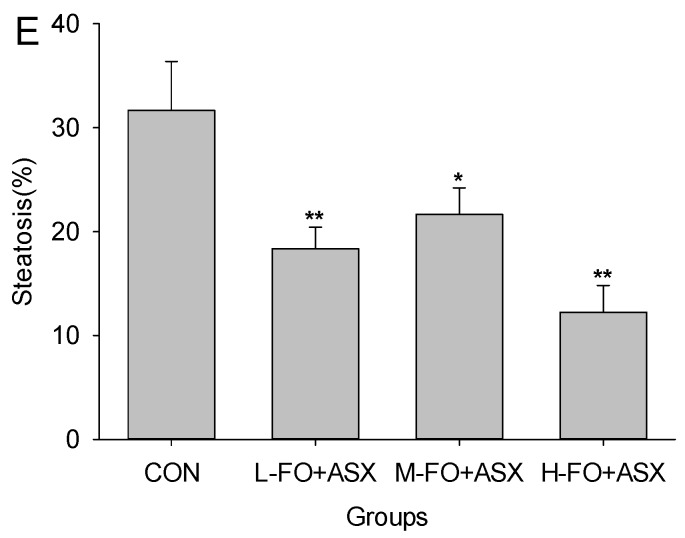
Liver histology after hematoxylin and eosin (H&E) staining of sections from representative rats from each group: (**A**) high-fat diet group; (**B**) low FO and ASX group; (**C**) moderate FO and ASX group and (**D**) high FO and ASX group. Arrow/arrowheads indicate micro- and macrovesicular steatosis, respectively. Asterisks mark scattered foci of macrovesicular steatosis; Volume density quantitation of hepatic steatosis (**E**). Bars represent means ± SEM (*n* = 5 animals/group). * *p* < 0.05 and ** *p* < 0.01 compared to CON group.

Photomicrographs of H&E-stained liver sections appear in Figure 2. Rats fed a high-fat diet for 10 weeks developed extensive microvesicular steatosis and scattered macrovesicular steatosis. However, when rats received FO + ASX, circular lipid droplets were reduced and even fewer were present in H-FO + ASX animals. Steatosis was less in all treated animals fed a high-fat diet as compared to controls.

### 3.4. Effects of FO and ASX on Liver Lipids

**Figure 3:**
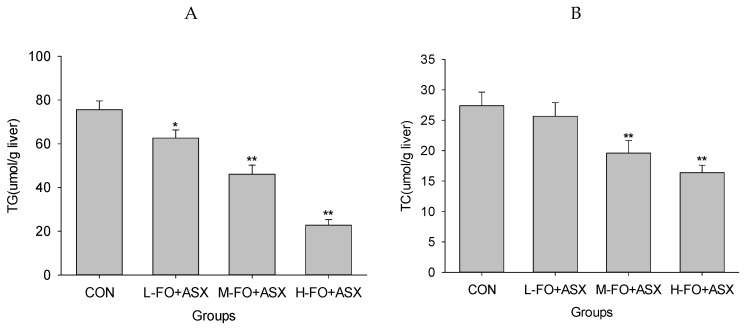
Effects of FO and ASX on hepatic triglyceride (TG) (**A**) and total cholesterol (TC) (**B**) in rats fed a high-fat diet. CON: high-fat diet group; L-, M- and H-FO + ASX: low, moderate, and high FO and ASX groups. Bars represent means ± SEM (*n* = 10 animals/group). * *p* < 0.05 and ** *p* < 0.01 compared to CON group.

As shown in Figure 3, hepatic TGs were lower in the L-FO + ASX, M-FO + ASX, and H-FO + ASX group than in controls. Similarly, livers from M-FO + ASX and H-FO + ASX animals had less TC compared to control rats.

### 3.5. Effects of FO and ASX on Liver Protein Expression

**Figure 4:**
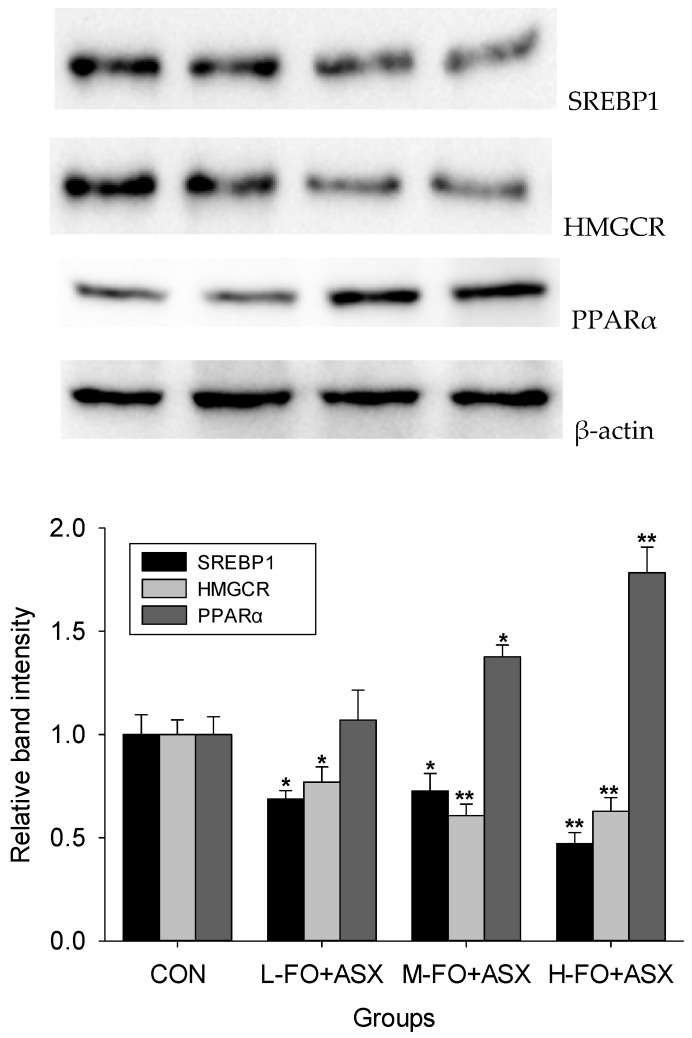
Effects of FO and ASX on hepatic lipid synthesis related proteins expression. Representative Immunoblot comparing relative SREBP1, HMGCR and PPARα protein. CON: high-fat diet group; L-, M- and H-FO + ASX: low, moderate, and high FO and ASX groups. Bars represent means ± SEM from 4 independent experiments. * *p* < 0.05 and ** *p* < 0.01 compared to CON group.

Compared to controls (Figure 4), all FO + ASX had less SREBP1 and HMGCR protein expression but more PPARα protein expression was observed in M-FO + ASX and H-FO + ASX groups.

### 3.6. Effects of FO and ASX on Liver mRNA Expression

**Figure 5:**
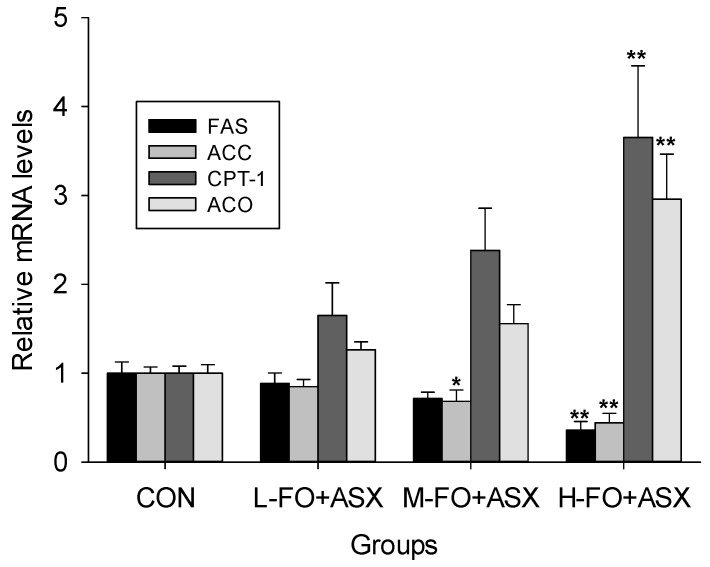
Effects of FO and ASX on hepatic FAS, ACC, CPT-1 and ACO mRNA. CON: high-fat diet group; L-, M- and H-FO + ASX: low, moderate, and high FO and ASX groups. Bars represent means ± SEM from 4 independent experiments. * *p* < 0.05 and ** *p* < 0.01 compared to CON group.

FAS and ACC, two important SREBP-1 target enzymes [31], are essential for fatty acid synthesis. Figure 5 show that FAS mRNA expression in the H-FO + ASX group and ACC in the M-FO + ASX and H-FO + ASX groups were lower than in controls. Conversely, H-FO + ASX significantly increased CPT-1 and ACO mRNA expression; these are PPAR α-induced rate-limiting enzymes of fatty acid oxidation [32,33].

### 3.7. Effects of FO and ASX on Liver Antioxidant Capacity and Lipid Peroxidation

**Figure 6:**
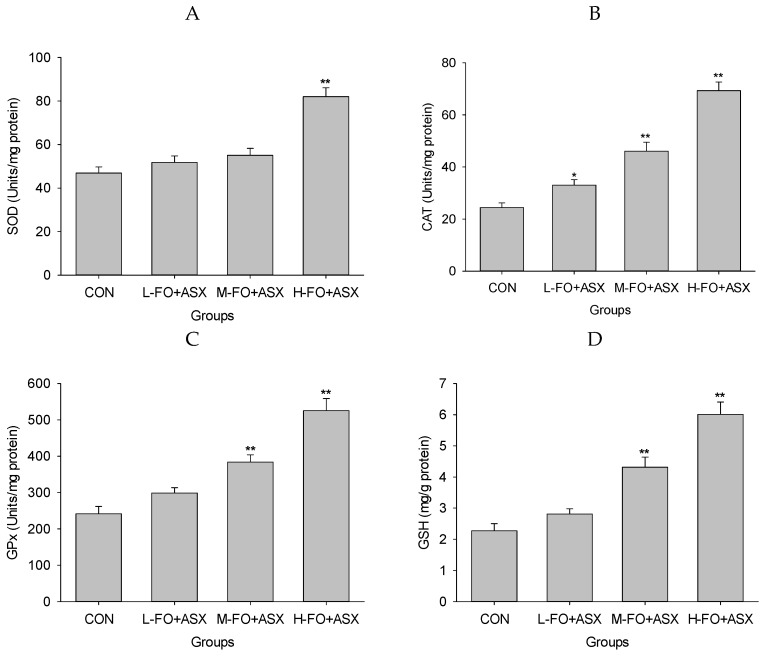

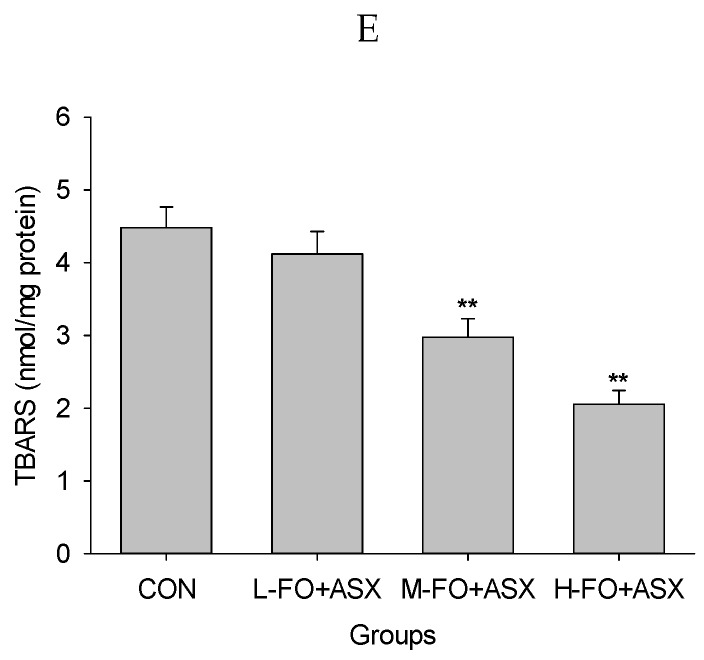
Effects of FO and ASX on SOD (**A**), GPx (**B**) and CAT activity (**C**), GSH (**D**), and TBARS (**E**) in liver of rats fed a high-fat diet. CON: high-fat diet group; L-, M- and H-FO + ASX: low, moderate, and high FO and ASX groups. Bars represent means ± SEM (*n* = 10 animals/group). * *p* < 0.05 and ** *p* < 0.01 compared to CON group.

Figure 6 shows that SOD activity in the H-FO + ASX group was increased compared to controls, as was CAT activity in all FO + ASX groups. GPx activity was greater in the M-FO + ASX and H-FO + ASX groups. After M-FO + ASX and H-FO + ASX treatment, GSH was elevated compared with controls. TBARS were lower in the M-FO + ASX and H-FO + ASX groups compared to controls.

## 4. Discussion

Much progress has been made to elucidate the pathogenesis of NAFLD. The most widely accepted mechanism for NAFLD is a “two-hit” theory [5]: the first hit being excessive hepatic fat accumulation which develops when an imbalance in fatty acid uptake and de novo lipogenesis exceeds oxidation and exportation [34]. The second hit is a hepatocellular injury due to oxidative stress, which drives the disease development. A high-fat diet induces significant lipid accumulation and oxidative stress [35], and may cause many chronic metabolic diseases [36] including NAFLD [37]. Liver histological analysis is used to evaluate the presence and severity of NAFLD, and this was used to show that a high-fat diet caused noticeable hepatic steatosis, the earliest stage of NAFLD. FO + ASX treatment diminished the severity of hepatic steatosis, and M- and H-FO + ASX significantly reduced hepatic triacylglycerol and cholesterol. Thus, reducing hepatic lipid accumulation is key (an important mechanism) for the combination of FO and ASX to reversing initial NAFLD. ALA can reduce fatty acid synthesis by suppressing numerous lipogenic enzymes, such as FAS and lipogenic transcriptional factor SREBP1 [38,39]. PPARα is needed to regulate fatty acid metabolism, and ALA is a natural ligand of PPARα, binding to and activating this critical transcriptional regulator to increase gene expression and enzyme activity involved in hepatic fatty acid oxidation [38,39,40,41,42]. ALA has more affinity for carnitine:palmitoyl transferase-1, the rate-limiting enzyme of fatty acid β-oxidation [43], which make ALA a preferential substrate relative to other fatty acids (such as 16:0 and 18:0 saturated fatty acids, oleic acid, linoleic acid) for mitochondrial and peroxisomal β-oxidation pathways [42,44]. Thus, the special spatial arrangement is determined by the number and position of double bonds [43], the greater extent of partitioning of ALA towards *β*-oxidation rather than deposition of TG. Depletion of hepatic *n*-3 long-chain PUFAs (LCPUFA) should favor TG synthesis over oxidation and may contribute to steatosis of NAFLD [45]. As the precursor fatty acid of *n*-3 LCPUFA, ALA significantly elevates EPA and DHA in hepatic membranes and reduces hepatic TG [46]. In addition, ALA increases CYP7A1 activity, which enhances secretion of hepatic cholesterol into bile and lowers hepatic cholesterol [47]. Similar to other antioxidant nutraceuticals [48], ASX can regulate liver lipid content. Peroxisome proliferator-activated receptor-γ (PPARγ) is thought to be a presteatotic factor in fatty liver [49]. ASX induces effects opposite to that of PPARα (of which it is an agonist) and PPARγ (of which it is an antagonist) [50], which has an overall effect of reducing hepatic lipid accumulation [51,52] by rewiring hepatic lipid metabolic gene expression [50,53]. Moreover, CYP27A1 regulates bile acid synthesis, and ASX can substantially induce CYP27A1 gene expression [50] and maintain hepatic cholesterol homeostasis. FO and ASX decrease hepatic expression of SREBP1 and HMGCR, but increased expression of PPARα. In terms of results, this combination potently depressed FAS and ACC expression and significantly induced CPT-1 and ACO expression. Thus, the combination of FO and ASX may decrease hepatic triacylglycerol and cholesterol by suppressing hepatic lipogenesis and cholesterol synthesis as well as by promoting lipid oxidation.

Data show that high-fat diet diminishes hepatic antioxidant status and elevates oxidative stress and lipid peroxidation [54,55]. After a FO + LA diet, H-FO + ASX treated animals had more SOD, CAT and GPx activity, and more GSH. More antioxidant activity and reduced lipid peroxidation suggested alleviation of hepatic oxidative stress. Although FO may reduce hepatic GSH depletion by lowering cholesterol and triacylglycerol [9], the potent antioxidant properties of ASX should be mainly responsible for the dramatic hepatic oxidative stress improvement effect after FO + LA consumption. Due to polar moieties on both ends of the polyene chain, ASX had more free radical scavenging capability than α-tocopherol or β-carotene [56,57,58]. Also, structurally damaged mitochondria is a major source of oxidative stress in NAFLD [59], and ASX protects the mitochondrial redox state and its functional integrity [60] to reduce ROS production. In addition, ASX can elevate hepatic antioxidant SOD, CAT and GPx expression and activity by inducing, at least partly, the Nrf2 pathway [52,53] and many non-enzymatic antioxidants such as GSH, vitamins E and C after a high-fat diet or other pathological conditions [16,52,61].

## 5. Conclusions

FO plus ASX can reduce hepatic steatosis, TG, cholesterol and oxidative stress, suggesting that this may reduce NAFLD induced by a high-fat diet. Thus, FO + ASX may be promising for hepatoprotection but more work is required in additional NAFLD models to confirm this hypothesis.

## Figures and Tables

**Table 1:** Nutrient content and ingredient composition of the experimental diets.

Diet	g	kcal
**Nutrient,%**		
Protein	20	17
Carbohydrate	50	43
Fat	20	39
Total		100
**Ingredient**		
Casein	200	800
dl-methionine	3	12
Maize starch	350	1400
Sucrose	150	600
Cellulose	50	0
Mineral mixture (AIN-93M)	35	0
Vitamin mixture (AIN-93M)	10	40
Choline bitartrate	2	0
Fat	200	1800
Total	1000	4652

## References

[1] Vuppalanchi R., Chalasani N. (2009). Nonalcoholic fatty liver disease and nonalcoholic steatohepatitis: Selected practical issues in their evaluation and management. Hepatology.

[2] Anderson N., Borlak J. (2008). Molecular mechanisms and therapeutic targets in steatosis and steatohepatitis. Pharmacol. Rev..

[3] Amarapurkar D., Kamani P., Patel N., Gupte P., Kumar P., Agal S., Baijal R., Lala S., Chaudhary D., Deshpande A. (2007). Prevalence of non-alcoholic fatty liver disease: Population based study. Ann. Hepatol..

[4] Ciccone M.M., Principi M., Ierardi E., Di Leo A., Ricci G., Carbonara S., Gesualdo M., Devito F., Zito A., Cortese F. (2015). Inflammatory bowel disease, liver diseases and endothelial function: Is there a linkage?. J. Cardiovasc. Med. (Hagerstown).

[5] Day C.P., James O.F. (1998). Steatohepatitis: A tale of two “hits”?. Gastroenterology.

[6] Anderson B.M., Ma D.W. (2009). Are all *n*-3 polyunsaturated fatty acids created equal?. Lipids Health Dis..

[7] Holman R.T. (1986). Control of polyunsaturated acids in tissue lipids. J. Am. Coll. Nutr..

[8] Barcelo-Coblijn G., Murphy E.J. (2009). Alpha-linolenic acid and its conversion to longer chain *n*-3 fatty acids: Benefits for human health and a role in maintaining tissue *n*-3 fatty acid levels. Prog. Lipid Res..

[9] Yang S.F., Tseng J.K., Chang Y.Y., Chen Y.C. (2009). Flaxseed oil attenuates nonalcoholic fatty liver of hyperlipidemic hamsters. J. Agric. Food Chem..

[10] Trebušak T., Levart A., Voljč M., Tomažin U., Pirman T. (2011). The effect of linseed oil supplementation on performance, fatty acid composition and oxidative status of rabbits. Acta Agric. Slov..

[11] Hussein G., Sankawa U., Goto H., Matsumoto K., Watanabe H. (2006). Astaxanthin, a carotenoid with potential in human health and nutrition. J. Nat. Prod..

[12] Hussein G., Nakamura M., Zhao Q., Iguchi T., Goto H., Sankawa U., Watanabe H. (2005). Antihypertensive and neuroprotective effects of astaxanthin in experimental animals. Biol. Pharm. Bull..

[13] Fassett R.G., Coombes J.S. (2011). Astaxanthin: A potential therapeutic agent in cardiovascular disease. Mar. Drugs.

[14] Ambati R.R., Phang S.M., Ravi S., Aswathanarayana R.G. (2014). Astaxanthin: Sources, extraction, stability, biological activities and its commercial applications—A review. Mar. Drugs.

[15] Ciccone M.M., Cortese F., Gesualdo M., Carbonara S., Zito A., Ricci G., De Pascalis F., Scicchitano P., Riccioni G. (2013). Dietary intake of carotenoids and their antioxidant and anti-inflammatory effects in cardiovascular care. Mediat. Inflamm..

[16] Bhuvaneswari S., Arunkumar E., Viswanathan P., Anuradha C.V. (2010). Astaxanthin restricts weight gain, promotes insulin sensitivity and curtails fatty liver disease in mice fed a obesity-promoting diet. Process Biochem..

[17] Bhuvaneswari S., Yogalakshmi B., Sreeja S., Anuradha C.V. (2014). Astaxanthin reduces hepatic endoplasmic reticulum stress and nuclear factor-kappab-mediated inflammation in high fructose and high fat diet-fed mice. Cell Stress Chaperones.

[18] Turkez H., Geyikoglu F., Yousef M.I., Togar B., Gurbuz H., Celik K., Akbaba G.B., Polat Z. (2014). Hepatoprotective potential of astaxanthin against 2,3,7,8-tetrachlorodibenzo-p-dioxin in cultured rat hepatocytes. Toxicol. Ind. Health.

[19] Takitani K., Miyazaki H., Yoden A., Tamai H. (2009). Children's toxicology from bench to bed—Liver injury (2): Mechanism of antioxidant therapy for nonalcoholic fatty liver disease. J. Toxicol. Sci..

[20] Xu J., Gao H., Zhang L., Chen C., Yang W., Deng Q., Huang Q., Huang F. (2014). A combination of flaxseed oil and astaxanthin alleviates atherosclerosis risk factors in high fat diet fed rats. Lipids Health Dis..

[21] Catta-Preta M., Mendonca L.S., Fraulob-Aquino J., Aguila M.B., Mandarim-de-Lacerda C.A. (2011). A critical analysis of three quantitative methods of assessment of hepatic steatosis in liver biopsies. Virchows Arch..

[22] Folch J., Lees M., Sloane Stanley G.H. (1957). A simple method for the isolation and purification of total lipides from animal tissues. J. Biol. Chem..

[23] Livak K.J., Schmittgen T.D. (2001). Analysis of relative gene expression data using real-time quantitative pcr and the 2(-delta delta c(t)) method. Methods.

[24] Kono Y. (1978). Generation of superoxide radical during autoxidation of hydroxylamine and an assay for superoxide dismutase. Arch. Biochem. Biophys..

[25] Sazuka Y., Tanizawa H., Takino Y. (1989). Effect of adriamycin on the activities of superoxide dismutase, glutathione peroxidase and catalase in tissues of mice. Jpn. J. Cancer Res..

[26] Goth L. (1991). A simple method for determination of serum catalase activity and revision of reference range. Clin. Chim. Acta.

[27] Moron M.S., Depierre J.W., Mannervik B. (1979). Levels of glutathione, glutathione reductase and glutathione s-transferase activities in rat lung and liver. Biochim. Biophys. Acta.

[28] Buege J.A., Aust S.D. (1978). Microsomal lipid peroxidation. Methods Enzymol..

[29] Xu J., Zhou X., Deng Q., Huang Q., Yang J., Huang F. (2011). Rapeseed oil fortified with micronutrients reduces atherosclerosis risk factors in rats fed a high-fat diet. Lipids Health Dis..

[30] Lowry O.H., Rosebrough N.J., Farr A.L., Randall R.J. (1951). Protein measurement with the folin phenol reagent. J. Biol. Chem..

[31] Shimano H. (2000). Sterol regulatory element-binding protein-1 as a dominant transcription factor for gene regulation of lipogenic enzymes in the liver. Trends Cardiovasc. Med..

[32] Clemenz M., Frost N., Schupp M., Caron S., Foryst-Ludwig A., Bohm C., Hartge M., Gust R., Staels B., Unger T. (2008). Liver-specific peroxisome proliferator-activated receptor alpha target gene regulation by the angiotensin type 1 receptor blocker telmisartan. Diabetes.

[33] Hashimoto T., Fujita T., Usuda N., Cook W., Qi C., Peters J.M., Gonzalez F.J., Yeldandi A.V., Rao M.S., Reddy J.K. (1999). Peroxisomal and mitochondrial fatty acid beta-oxidation in mice nullizygous for both peroxisome proliferator-activated receptor alpha and peroxisomal fatty acyl-coa oxidase. Genotype correlation with fatty liver phenotype. J. Biol. Chem..

[34] Musso G., Gambino R., Cassader M. (2009). Recent insights into hepatic lipid metabolism in non-alcoholic fatty liver disease (nafld). Prog. Lipid Res..

[35] Lieber C.S., Leo M.A., Mak K.M., Xu Y., Cao Q., Ren C., Ponomarenko A., DeCarli L.M. (2004). Model of nonalcoholic steatohepatitis. Am. J. Clin. Nutr..

[36] Heinonen I., Rinne P., Ruohonen S.T., Ruohonen S., Ahotupa M., Savontaus E. (2014). The effects of equal caloric high fat and western diet on metabolic syndrome, oxidative stress and vascular endothelial function in mice. Acta Physiol..

[37] Omagari K., Kato S., Tsuneyama K., Inohara C., Kuroda Y., Tsukuda H., Fukazawa E., Shiraishi K., Mune M. (2008). Effects of a long-term high-fat diet and switching from a high-fat to low-fat, standard diet on hepatic fat accumulation in sprague-dawley rats. Dig. Dis. Sci..

[38] Devarshi P.P., Jangale N.M., Ghule A.E., Bodhankar S.L., Harsulkar A.M. (2013). Beneficial effects of flaxseed oil and fish oil diet are through modulation of different hepatic genes involved in lipid metabolism in streptozotocin-nicotinamide induced diabetic rats. Genes Nutr..

[39] Ide T., Kobayashi H., Ashakumary L., Rouyer I.A., Takahashi Y., Aoyama T., Hashimoto T., Mizugaki M. (2000). Comparative effects of perilla and fish oils on the activity and gene expression of fatty acid oxidation enzymes in rat liver. Biochim. Biophys. Acta.

[40] Ide T. (2000). Effect of dietary alpha-linolenic acid on the activity and gene expression of hepatic fatty acid oxidation enzymes. BioFactors.

[41] Murase T., Aoki M., Tokimitsu I. (2005). Supplementation with alpha-linolenic acid-rich diacylglycerol suppresses fatty liver formation accompanied by an up-regulation of beta-oxidation in zucker fatty rats. Biochim. Biophys. Acta.

[42] Ide T., Murata M., Sugano M. (1996). Stimulation of the activities of hepatic fatty acid oxidation enzymes by dietary fat rich in alpha-linolenic acid in rats. J. Lipid Res..

[43] Clouet P., Niot I., Bezard J. (1989). Pathway of alpha-linolenic acid through the mitochondrial outer membrane in the rat liver and influence on the rate of oxidation. Comparison with linoleic and oleic acids. Biochem. J..

[44] DeLany J.P., Windhauser M.M., Champagne C.M., Bray G.A. (2000). Differential oxidation of individual dietary fatty acids in humans. Am. J. Clin. Nutr..

[45] Videla L.A., Rodrigo R., Araya J., Poniachik J. (2004). Oxidative stress and depletion of hepatic long-chain polyunsaturated fatty acids may contribute to nonalcoholic fatty liver disease. Free Radic. Biol. Med..

[46] Kim H.K., Choi H. (2001). Dietary alpha-linolenic acid lowers postprandial lipid levels with increase of eicosapentaenoic and docosahexaenoic acid contents in rat hepatic membrane. Lipids.

[47] Morise A., Serougne C., Gripois D., Blouquit M.F., Lutton C., Hermier D. (2004). Effects of dietary alpha linolenic acid on cholesterol metabolism in male and female hamsters of the lpn strain. J. Nutr. Biochem..

[48] Scicchitano P., Cameli M., Maiello M., Modesti P.A., Muiesan M.L., Novo S., Palmiero P., Saba P.S., Pedrinelli R., Ciccone M.M. (2014). Nutraceuticals and dyslipidaemia: Beyond the common therapeutics. J. Funct. Foods.

[49] Moran-Salvador E., Lopez-Parra M., Garcia-Alonso V., Titos E., Martinez-Clemente M., Gonzalez-Periz A., Lopez-Vicario C., Barak Y., Arroyo V., Claria J. (2011). Role for ppargamma in obesity-induced hepatic steatosis as determined by hepatocyte- and macrophage-specific conditional knockouts. FASEB J..

[50] Jia Y., Kim J.Y., Jun H.J., Kim S.J., Lee J.H., Hoang M.H., Hwang K.Y., Um S.J., Chang H.I., Lee S.J. (2012). The natural carotenoid astaxanthin, a ppar-alpha agonist and ppar-gamma antagonist, reduces hepatic lipid accumulation by rewiring the transcriptome in lipid-loaded hepatocytes. Mol. Nutr. Food Res..

[51] Ikeuchi M., Koyama T., Takahashi J., Yazawa K. (2007). Effects of astaxanthin in obese mice fed a high-fat diet. Biosci. Biotechnol. Biochem..

[52] Yang Y., Seo J.M., Nguyen A., Pham T.X., Park H.J., Park Y., Kim B., Bruno R.S., Lee J. (2011). Astaxanthin-rich extract from the green alga haematococcus pluvialis lowers plasma lipid concentrations and enhances antioxidant defense in apolipoprotein e knockout mice. J. Nutr..

[53] Yang Y., Pham T.X., Wegner C.J., Kim B., Ku C.S., Park Y.K., Lee J.Y. (2014). Astaxanthin lowers plasma tag concentrations and increases hepatic antioxidant gene expression in diet-induced obesity mice. Br. J. Nutr..

[54] Jadeja R.N., Thounaojam M.C., Dandekar D.S., Devkar R.V., Ramachandran A.V. (2010). Clerodendron glandulosum.Coleb extract ameliorates high fat diet/fatty acid induced lipotoxicity in experimental models of non-alcoholic steatohepatitis. Food Chem. Toxicol..

[55] Raso G.M., Esposito E., Iacono A., Pacilio M., Cuzzocrea S., Canani R.B., Calignano A., Meli R. (2009). Comparative therapeutic effects of metformin and vitamin E in a model of non-alcoholic steatohepatitis in the young rat. Eur. J. Pharmacol..

[56] Shimidzu N., Goto M., Miki W. (1996). Carotenoids as singlet oxygen quenchers in marine organisms. Fish. Sci..

[57] Krinsky N.I. (1989). Antioxidant functions of carotenoids. Free Radic. Biol. Med..

[58] Miki W. (1991). Biological functions and activities of animal carotenoids. Pure Appl. Chem..

[59] McCarty M.F. (2011). Full-spectrum antioxidant therapy featuring astaxanthin coupled with lipoprivic strategies and salsalate for management of non-alcoholic fatty liver disease. Med. Hypotheses.

[60] Wolf A.M., Asoh S., Hiranuma H., Ohsawa I., Iio K., Satou A., Ishikura M., Ohta S. (2010). Astaxanthin protects mitochondrial redox state and functional integrity against oxidative stress. J. Nutr. Biochem..

[61] Sangeetha R.K., Baskaran V. (2010). Retinol-deficient rats can convert a pharmacological dose of astaxanthin to retinol: Antioxidant potential of astaxanthin, lutein, and beta-carotene. Can. J. Physiol. Pharmacol..

